# Assessment of traumatic mandibular nerve using MR neurography sequence: a preliminary study

**DOI:** 10.1186/s12903-024-04514-0

**Published:** 2024-06-28

**Authors:** Hyunwoo Yang, Nak-hoon Son, Dongwook Kim, Jae-Hee Chun, Jin Sung Kim, Tae Kyung Oh, Minwook Lee, Hyung Jun Kim

**Affiliations:** 1 Department of Oral and Maxillofacial Surgery, Yonsei University College of Dentistry, 50-1 Yonsei-ro, Seodaemun-gu, Seoul, 03722 Republic of Korea; 2 Department of Statistics, Keimyung University, Daegu, Republic of Korea; 3 Department of Radiation Oncology, Yonsei Cancer Center, Yonsei University College of Medicine, Seoul, Republic of Korea; 4 Department of Radiology, Yonsei University College of Medicine, Seoul, Republic of Korea

**Keywords:** MR neurography, Linear model, Signal intensity, Dental implant, Mandibular nerve, Inferior alveolar nerve, Lingual nerve

## Abstract

**Background:**

Iatrogenic mandibular nerve damage resulting from oral surgeries and dental procedures is painful and a formidable challenge for patients and oral surgeons alike, mainly because the absence of objective and quantitative methods for diagnosing nerve damage renders treatment and compensation ambiguous while often leading to medico-legal disputes. The aim of this study was to examine discriminating factors of traumatic mandibular nerve within a specific magnetic resonance imaging (MRI) protocol and to suggest tangible diagnostic criteria for peripheral trigeminal nerve injury.

**Methods:**

Twenty-six patients with ipsilateral mandibular nerve trauma underwent T2 Flex water, 3D short tau inversion recovery (STIR), and diffusion-weighted imaging (DWI) acquired by periodically rotating overlapping parallel lines with enhanced reconstruction (PROPELLER) pulse sequences; 26 injured nerves were thus compared with contra-lateral healthy nerves at anatomically corresponding sites. T2 Flex apparent signal to noise ratio (FSNR), T2 Flex apparent nerve-muscle contrast to noise ratio (FNMCNR) 3D STIR apparent signal to noise ratio (SSNR), 3D STIR apparent nerve-muscle contrast to noise ratio (SNMCNR), apparent diffusion coefficient (ADC) and area of cross-sectional nerve (Area) were evaluated.

**Results:**

Mixed model analysis revealed FSNR and FNMCNR to be the dual discriminators for traumatized mandibular nerve (*p* < 0.05). Diagnostic performance of both parameters was also determined with area under the receiver operating characteristic curve (AUC for FSNR = 0.712; 95% confidence interval [CI]: 0.5660, 0.8571 / AUC for FNMCNR = 0.7056; 95% confidence interval [CI]: 1.011, 1.112).

**Conclusions:**

An increase in FSNR and FNMCNR within our MRI sequence seems to be accurate indicators of the presence of traumatic nerve. This prospective study may serve as a foundation for sophisticated model diagnosing trigeminal nerve trauma within large patient cohorts.

**Supplementary Information:**

The online version contains supplementary material available at 10.1186/s12903-024-04514-0.

## Introduction

The mandibular nerve, which is the third branch of the trigeminal nerve, often suffers damage due to iatrogenic causes such as dental procedures and maxillofacial surgeries, presenting an excruciating experience for both patients and clinicians. The reported prevalence of these injuries varies in the literature; however, according to a specific study, nearly all practitioners (94.5%) experienced some cases of iatrogenic mandibular nerve injury within a 12-month period [1]. Inferior alveolar nerve (IAN) and lingual nerve (LN), peripheral branches of mandibular nerve, are most commonly affected due to their close proximity to dental surgery [2, 3]. Symptoms starting from mild hypoesthesia to frustrating paresthesia, excruciating dysesthesia and even complete anesthesia may arise on innervated sensory areas of IAN and LN. Functional abnormalities such as biting of the cheeks, drooling of saliva and fluids when eating, and difficulty in speech may also occur along with the above-mentioned sensory deficiencies. Most dire, however, is the chronological exacerbation of reactive depression on top of these symptoms, frequently followed by medico-legal controversy. Despite the histological classification of nerve injuries, first established by Seddon [4] in 1943, and later revised by Sunderland [5] in 1951, clinical correlation and subsequent diagnosis has mainly relied upon clinical neurosensory tests (NSTs) [6] or the Medical Research Council Scale (MRCS) [7]. These tests and scales, however, are based on patient response to stimuli and operator experience and interpretation, which are often semi-quantitative and subjective. To overcome such limitations of traditional methods, researchers began to utilize high resolution MR imaging to objectively evaluate peripheral trigeminal neuropathies, and in particular to define an MRI sequence which visualizes peripheral nerves with high contrast and resolution known as MR neurography [8–10]. Although various MRI sequences have been attempted to visualize peripheral trigeminal nerves and valuable MRI-based data has been used to characterize peripheral trigeminal neuropathies, no gold standard nor instinctive discriminator has yet been established. This prospective study aims to identify visible discriminators for traumatic mandibular nerve injuries by combining mixed model analysis and logistic regression for the first time. Additionally, we seek to determine the clinical applicability of this approach by integrating imaging parameters from three different MRI sequences with clinical information.

## Materials and methods

### Study design

This prospective MRI study was conducted at the Department of Oral and Maxillofacial Surgery, Yonsei University. Twenty-six patients (4 males, 22 females; overall age range 21–79 years; overall mean age and standard deviation 56 ± 23 years) suffering from unilateral traumatic mandibular nerve injury due to extraction or implant surgery were included in the study from April 2020 until April 2021. Patient information was collected and trigeminal nerve neuropathy diagnosis based on NSTs and MRCS was performed by two board-certified oral and maxillofacial surgeons (H.J.K. and D.W.K.). Based on NSTs and clinical symptoms, symptom severity was categorized according to MRCS. Briefly, grades of MRCS are as follows: S0 (no sensation), S1 (deep cutaneous pain in autonomous zone), S2 (some superficial pain and touch), S2+ (superficial pain and touch plus hyperesthesia), S3 (superficial pain and touch without hyperesthesia; static 2-point discrimination 15 mm), S3+ (the same as S3 with good stimulus localization and static 2-point discrimination of 7–15 mm), S4 (the same as S3 and static 2-point discrimination of 2–6 mm) [7]. Patients with S0/S1 were classified as severe, S2/S2 + moderate, and S3/S3+/S4 mild. Significant neuropathic pains such as paresthesia (an abnormal sensation, whether spontaneous or evoked), dysesthesia (an unpleasant abnormal sensation, whether spontaneous or evoked), allodynia (pain caused by a stimulus that does not normally provoke pain), and hyperalgesia (increased pain sensitivity and extreme response to pain) were recorded. Terms of neuropathic pains and their definitions were according to the Classification of Chronic Pain, revised by the Taxonomy Committee of the International Association for the Study of Pain (IASP) in 2012 (detailed information available on the IASP website: http://www.iasp-pain.org). Supplementary Table 1 presents overall patient clinico-pathologic information. We performed a quantitative sensory test (QST) of NST on all patients; the test included brush stroke perception, mechanical touch threshold and 2-point discrimination. However, the primary focus of this study was on examining the correlation between symptom severity and MR metrics. Specific NST results were omitted from this manuscript. Twenty-one IAN and five LN from the trauma sites were compared to sites on the corresponding contralateral side, which served as the control. Exclusion criteria were bilateral mandibular nerve trauma, patients with central trigeminal nerve neuropathy, and standard contraindications for MRI (e.g., implanted pacemaker). The trial was performed in accordance with ethical guidelines and received Institutional Review Board approval of the Yonsei University, Seoul Korea. (Approval number 2-2020-0019). Written informed consent was given by all subjects.

### MR Imaging

All subjects underwent MRI on a 3.0T system (Pioneer; GE Healthcare, Waukesha, WI, USA) using a 21-channel head coil. Subjects were positioned head-first in a supine position. Sequence specifications for the T2 Flex water, 3D STIR, and DWI PROPELLER are listed in Table 1. Along with the sequences, post-processing using maximum intensity projection (MIP) was performed to evaluate mandibular nerves along their trajectory. MIP was performed on a portion of the T2 sequence, and it was used for confirmation purposes in selecting ROIs when nerve tracing was ambiguous (Thickness: 3 mm).

**Table 1:** Parameters for the dedicated T2 flex water, 3D STIR and DWI propeller sequences of this study

Parameter	T2 Flex	3D CUBE STIR	DWI Propeller
TR [msec]	2763	1802	6553
TE [msec]	74.4	59.1	77
TI [msec]	N/A	210.0	N/A
Slice thickness [mm]	4.0	2.0	4.0
Intersection gap	2.0	-1.0 (overlapping)	1.5
FOV [cm]	25.0 × 22.5	24.0 × 24.0	25.0 × 25.0
Acquisition matrix	320 × 288	416 × 320	96 × 96
Time of acquisition [mm: ss]	~ 04:00	~ 04:00	~ 09:00
Number of excitations	1.0	1.0	2.5
Pixel bandwidth [Hz]	520.6	437.0	325.5
Flip angle	111	90	110.0
Echo train length	10	46	20
Diffusion B Value [s/mm^2^]	N/A	N/A	500

TR: repetition time; TE: time to echo; TI: time to inversion; FOV: field of view; STIR: short tau inversion recovery

### Image analysis

Each MRI image was normalized to have a similar contrast based on the average of the top 90% voxel values. All the analyses and evaluations in this study were conducted in MATLAB (The MathWorks, Inc.) using DICOM file information. All image data were anonymized before analysis.

The metrics applied to analyze the medical significance of injured trigeminal nerves were as follows: T2 Flex apparent signal to noise ratio (FSNR), apparent nerve-muscle contrast to noise ratio (FNMCNR) 3D STIR apparent signal to noise ratio (SSNR), 3D STIR apparent nerve-muscle contrast to noise ratio (SNMCNR), apparent diffusion coefficient (ADC), and area of cross-sectional nerve (Area). FSNR, FNMCNR, SSNR, and SNMCNR were determined using the coronal section of Flex and STIR images in axial reconstructions along their proximo-distal pathways. Meanwhile, ADC was measured at the same anatomical location where FSNR and SSNR were measured on axial images of the DWI sequence. To avoid possible interference by metallic artifacts (dental implants and prosthesis) in the nerve signal measurements, the region of interest (ROI) was placed approximately 1 cm proximal and 1 cm distal to the site of iatrogenic injury. The signal intensity (SI) of neural structures was quantified by delineating regions of interest (ROI) in both the proximal and distal segments of the inferior alveolar nerve (IAN) and lingual nerve (LN), as described in the preceding paragraph. To characterize the nerve-muscle contrast of the inferior alveolar nerve (IAN) and lingual nerve (LN), a region of interest (ROI) was positioned within ipsilateral masseter muscle. Subsequently, the respective values were obtained and incorporated into formulas as outlined by Klupp et al. [11] and applied within many other studies [12–14]:

FSNR : T2 Flex SInerve/T2 Flex SDnerve.

FNMCNR : T2 Flex (SInerve − Simuscle)/T2 Flex SDnerve.

SSNR : 3D STIR SInerve/3D STIR SDnerve.

SNMCNR : 3D STIR (SInerve − Simuscle)/3D STIR SDnerve.

**Fig. 1:**
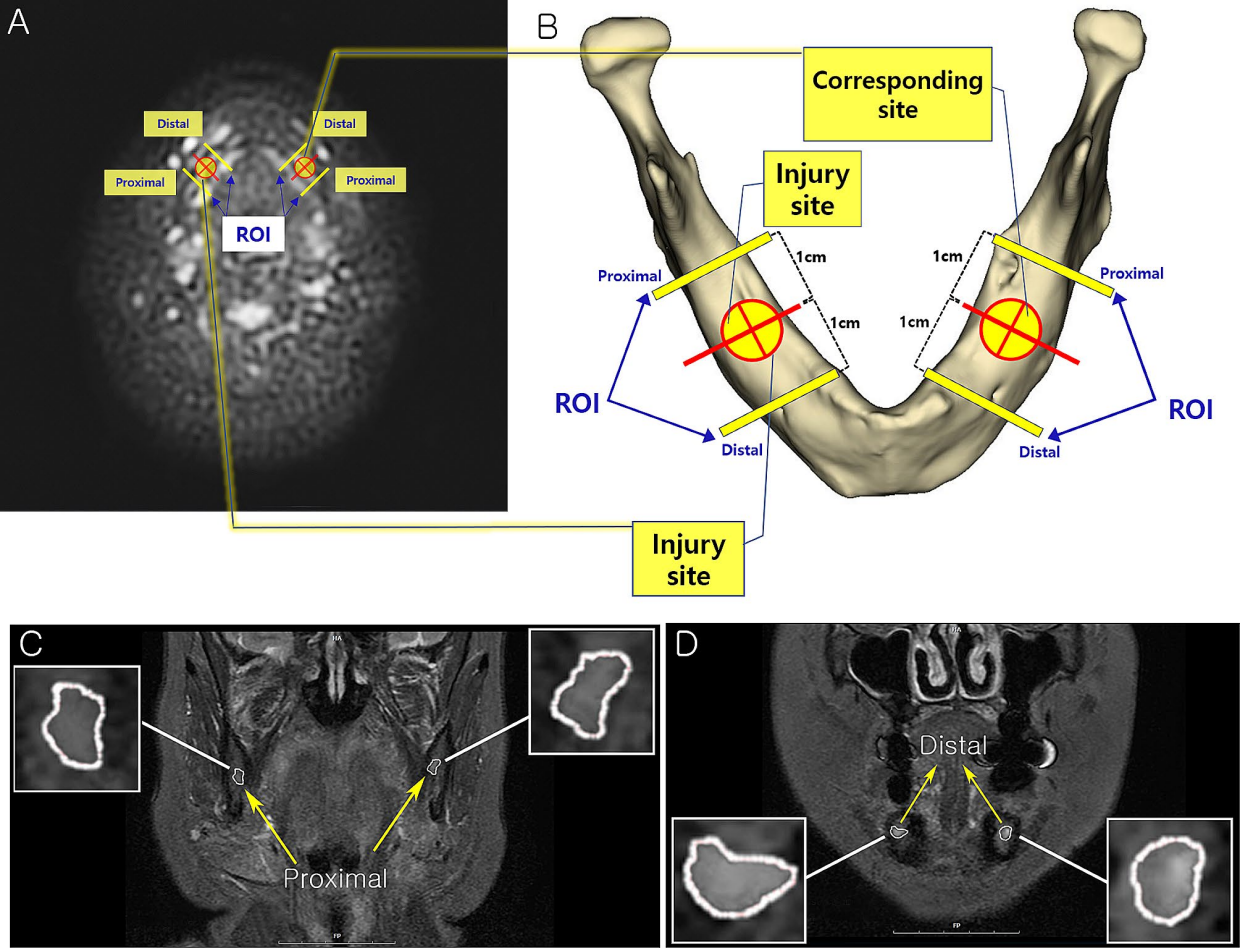
Schematic image of ROI selection and circumferential nerve delineation. (**A**) Axial view of DWI PROPELLER image (b = 500); (**B**) Schematic image of mandible illustrating the injury site and location of ROIs 1 cm apart from the site in proximo-distal aspect; (**C**) Coronal T2 Flex ROI of proximal level; (**D**) Coronal T2 Flex ROI of distal level; DWI, diffusion weighted imaging; PROPELLER, periodically rotating overlapping parallel lines with enhanced reconstruction; ROI, region of interest

Within the ROIs, the calculated metrics (FSNR, FNMCNR SSNR, FNMCNR, ADC and Area) were analyzed by a skilled medical physicist (J.H.C.). The ROIs were selected and circumferential nerve delineation were manually drawn by an oral and maxillofacial surgeon (H.Y.), then reaffirmed by a third-year oral and maxillofacial resident (T.K.O.). Furthermore, equal measures on the contralateral side were analyzed in the mandibular body at the same level as the trauma sites. Schematic images of plane selection analysis and circumferential nerve delineation are depicted in Fig. 1.

### Statistical analysis

To evaluate interobserver agreement between H.Y. and T.K.O. for nerve delineation on coronal sections of Flex and STIR images, as well as ROI selection on DWI axial images, we utilized the intraclass correlation coefficient (ICC). A correlation was considered good if the ICC value was ≥ 0.65, moderate for values from < 0.65 to ≥ 0.50, fair for values from < 0.50 to ≥ 0.40, and poor for values < 0.40.

In order to find a correlation between clinical degree of trauma (categorized as mild, moderate, and severe by MRSC) and significant imaging metrics (FSNR, FNMCNR, SSNR, SNMCNR, ADC and Area), Pearson’s and Spearman’s correlation analysis, ANOVA, and Kruskal-Wallis test were performed independently for trauma and normal sides. Furthermore, the correlation between trauma and normal side was statistically analyzed via Fisher’s Z transformation method to validate a significant MRI metric that accorded with clinical information. These statistical tests were two-sided, *p* values < 0.05 being considered statistically significant.

Furthermore, analysis was conducted to identify significant radiological parameters discriminating the site of injury. Both normal and traumatic sides were considered in one subject, each parameter being evaluated using a linear mixed model with correlated data (as observed at the same time in one person on the traumatic nerve and contra-lateral sides) in the MIXED procedure of SAS (Version 9.4; SAS Institute, Cary, NC) with a restricted maximum likelihood estimation. The analysis used the observed data from each patient with no imputation for missing data. The equation for the linear mixed model is as follows:

$${y}_{i}={x}_{i type}{\beta }_{i type}+{z}_{i}+ {\varepsilon }_{i}$$

Where $i$: subjects, $type$: independent variables (FSNR, FNMCNR, SSNR, SNMCNR ADC, and Area)

Meanwhile, since nerve damage was caused by external factors (traumatic dental treatment), the concept of mutual independence was applied as an alternative approach. Traumatic nerve side and normal side were considered independent and logistic regression analysis was performed; subsequently, the receiver operating characteristic (ROC) curve analysis was performed and the area under the curve (AUC) calculated.

## Results

### Inter-observer agreement on nerve delineation and ROI selection

Interobserver agreement was good for nerve delineation on coronal sections of Flex and STIR images (ICC of normal side: 0.81; ICC of trauma side: 0.82) and ROI selection on DWI axial images (ICC of normal side: 0.68; ICC of trauma side: 0.73).

### Correlation analysis between clinical degree and MR metrics

The correlation between imaging metrics (FSNR, FNMCNR, SSNR, SNMCNR ADC, and Area) and clinical degree of trauma could not be confirmed in spite of multiple correlation analyses (Pearson’s, Spearman’s correlation analysis, ANOVA, and Kruskal-Wallis test) and Fisher’s Z transformation method (Supplementary Tables 2–5).

### Mixed model analysis

Based on calculated metrics (FSNR, FNMCNR, SSNR, SNMCNR ADC, and Area), mixed model analysis was performed to investigate discriminators for traumatic nerve. As shown in Table 2, FSNR (*p* = 0.0243) and FNMCNR (*p* = 0.0152) were the dual factors associated with mandibular nerve injury.

**Table 2:** The Linear mixed model results

Variables	Estimate	SE	*P*-value
**FSNR**	0.005737	0.002394	**0.0243**
**FNMCNR**	0.009302	0.003079	**0.0057**
SSNR	0.02491	0.9618	0.9795
SNMCNR	0.005309	0.003340	0.1246
ADC	0.08699	0.1696	0.6124
Area	0.01028	0.01221	0.4077

FSNR: T2 Flex apparent signal to noise ratio; FNMCNR: T2 Flex apparent nerve-muscle contrast to noise ratio; SSNR: 3D STIR apparent signal to noise ratio; SNMCNR: 3D STIR apparent nerve-muscle contrast to noise ratio; ADC: Apparent diffusion coefficient; Area: Area of cross sectional nerve

### ROC analysis of diagnostic performance

**Fig. 2:**
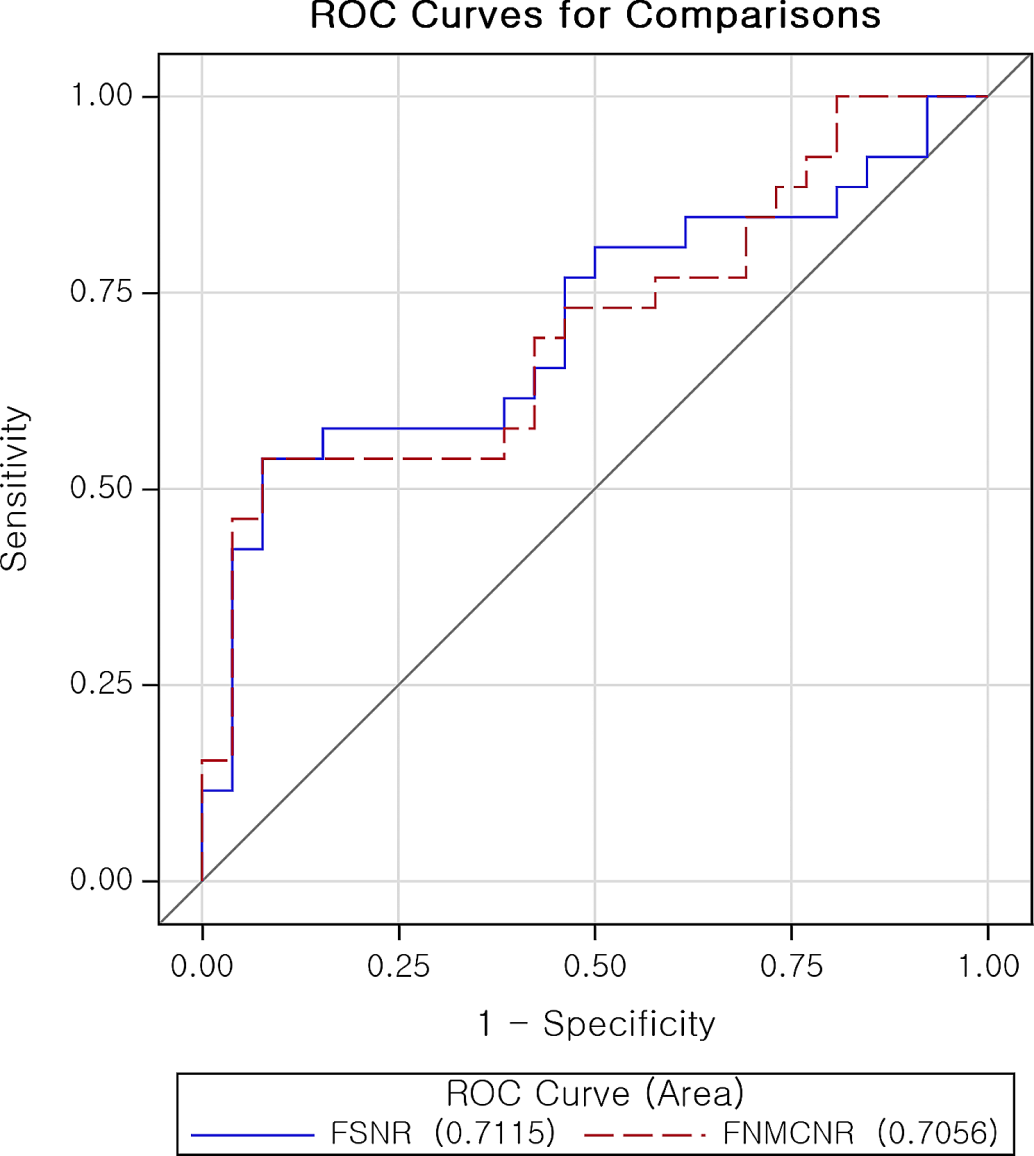
Diagnostic performance of FSNR and FNMCNR for traumatic nerve exhibited via ROC curve. (AUC for FSNR = 0.7115; 95% confidence interval [CI]: 0.5660, 0.8571); (AUC for FNMCNR = 0.7056; 95% confidence interval [CI]: 0.5610, 0.8503); AUC: Area under the receiver operating characteristic curve; FSNR: T2 Flex apparent signal to noise ratio; FNMCNR: T2 Flex apparent nerve-muscle contrast to noise ratio; ROC: Receiver operating characteristic

As a result of logistic regression, FSNR (*p*-value = 0.0401) and FNMCNR (*p*-value = 0.0152) were confirmed as a significant factor; Nerve T2 signal increase of Flex sequence determined traumatic peripheral nerve injury with reasonable diagnostic performance. The diagnostic performance of this imaging metric was obtained using area under the curve (AUC) as shown in Fig. 2; AUC = 0.7115 for FSNR, AUC = 0.7056 for FNMCNR).

## Discussion

This preliminary study demonstrates that specific parameters of MRI sequences (FSNR, FNMCNR) can discriminate traumatic conditions of the mandibular nerve. Simultaneously, it provides evidence of the considerable diagnostic performance of these variables, quantifying the feasibility of a visible, image-based diagnosis of traumatic mandibular nerve injuries.

Moreover, our study revealed the significance of signal intensity in diagnosing trigeminal peripheral nerve trauma via mixed model analysis. Although many other MR neurography studies agree with this concept, the uniqueness of this study lies in its study design [15–19]. At first, as in many other previous post-traumatic trigeminal neuropathy MR neurography studies, we regarded the trauma and contra-lateral normal sides as independent, logically expecting to find a significant correlation between clinical degree of trauma (categorized by MRSC) and potential MRI metrics (FSNR, FNMCNR, SSNR, SNMCNR, ADC and Area; Tables S2 - S5). To validate this hypothesis, Fisher’s Z transformation method was applied to each correlation analysis; however, statistical significance was not confirmed. Upon reflection, we attribute this to MRI imaging metrics being correlated data which reflect characteristics of each patient. Moreover, in this study they were simultaneously estimated based on both trauma and normal side. Therefore, regarding the affected and contra-lateral sides as independent when conducting statistical analysis may yield imprecise results. When a mixed effect model was used to analyze MR imaging metrics, as mentioned above, T2 Flex signal intensity (FSNR, FNMCNR) turned out to be dual discriminating factors for peripheral trigeminal nerve trauma. Logistic regression analysis also strongly supported the aforementioned mixed effect, yielding decent diagnostic performance as expressed in terms of AUC (Fig. 2).

Bioscience and medical research often require repeated measurement of targeted variables. In the present study, although FSNR, FNMCNR, SSNR, SNMCNR ADC, and Area are unique properties of each patient, cross-sectional images on which metrics were analyzed are drawn from four sites (Fig. 1), resulting in four values for each metric. Therefore, the process of repeated measurements should be considered. Many researchers often use Pearson’s or Spearman’s correlation coefficient to analyze the correlation between repeatedly measured variables without considering repeated measurements. The process of taking the total number of measurements into account increases the degree of freedom, resulting in undesirable type I error [20]. A few, researchers have tried to discern the diagnostic value of MR neurography in post-traumatic trigeminal nerve. Despite promising results, in the process of differentiating injured nerves from controls and finding correlations between MRN findings and clinical data, researchers often fail to address protocols for repeated measurements and ROI selection [17, 21, 22].

Another important factor to consider in designing a MR neurography study is proper control selection. Despite its numerous advantages, MRI has evident shortcomings, such as geometric distortions, signal dropout, and artifacts [23]. While signal dropout and artifacts can be managed by sequence modification, geometric distortion, especially when patient-dependent, is very difficult to correct [24]. Geometric distortion affects the original images, which may in turn affect the delineation of target morphology and clinical target volume, thus ultimately affecting the diagnosis [23] Enrolling MRI images of healthy volunteers to create reference targets must therefore be done with caution, even under the same MR sequences. The above-mentioned reasons statistically and medico-physically support applying linear mixed model analysis and not enrolling a healthy control group.

**Fig. 3:**
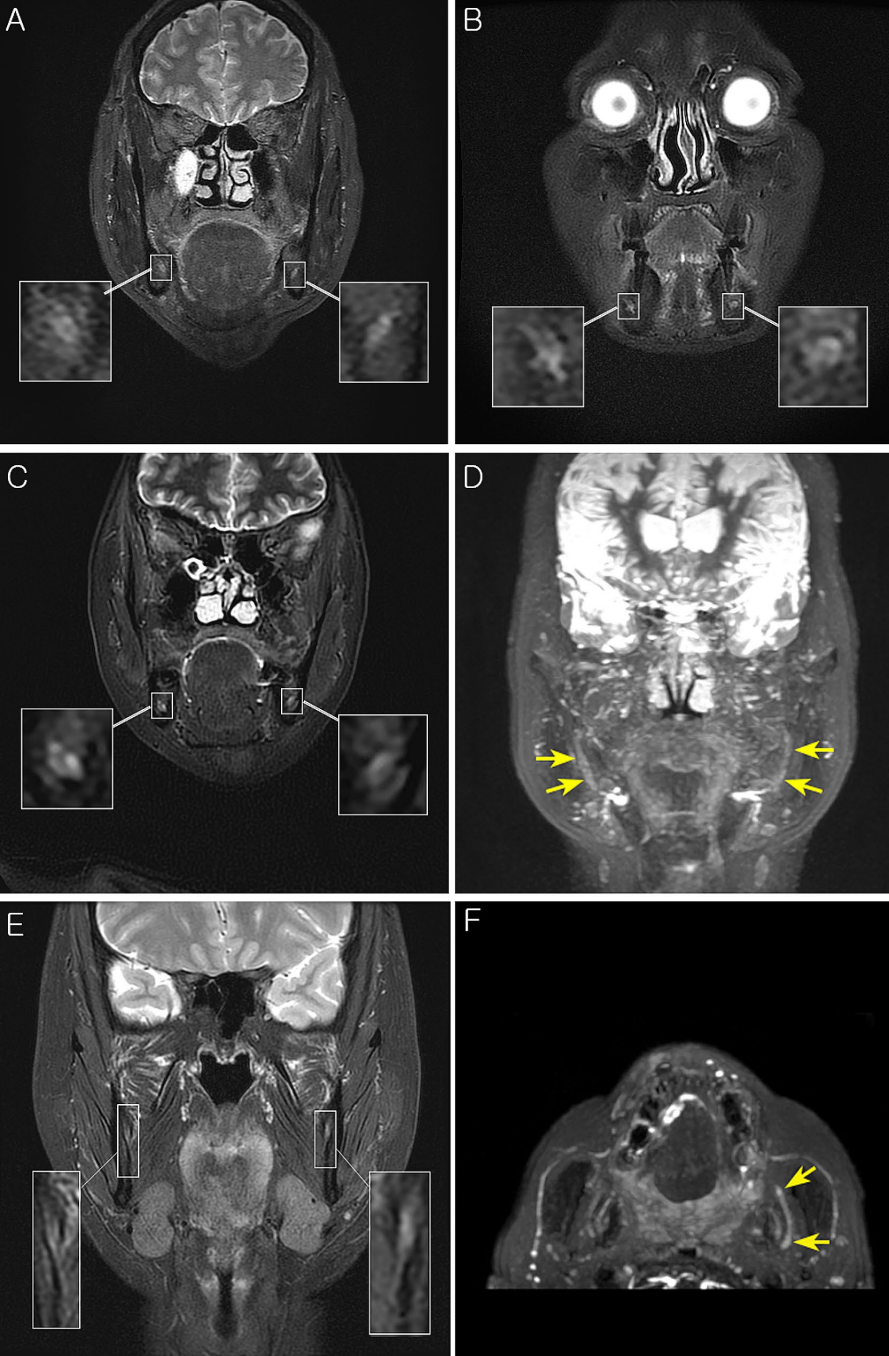
Example MR images demonstrating features of present study. (**A-B**) Representative high contrast MR images with sufficient resolution to demonstrate nerve fascicles; (**C**) Representative cross-sectional view demonstrating higher signal of trauma side (Right IAN); (**D**) Maximum intensity projection of (**C**) also demonstrates asymmetric hyperintensity of traumatized (right) nerve; (**E**) Representative cross-sectional T2 Flex image including the ROI of the traumatic left lingual nerve; (**F**) Maximum intensity projection of (**E**) demonstrating ‘Triple B’ sign of left lingual nerve (arrow)

Several factors render MR neurography more difficult to implement, especially in the head and neck region. First, the cranial nerves, including trigeminal nerves, are of small caliber and follow a tortuous course, penetrating tissues with very different physical properties. The melting pot of various anatomic structures of head and neck region requires more stable and functional sequences. Ideally, a composed cranial nerve MR neurography sequence includes a large field of view (FOV) with thin slice thickness, high signal and contrast-to-noise ratios, uniform fat, venous and arterial suppression, and minimal magic angle artifacts. All these requirements should be met within reasonable acquisition times and with minimum chance for motion artifacts [25]. To obtain optimum MR neurography images in a limited scan time, the present study utilized both Flex (Dixon technique) and STIR methods based on a T2-weighted sequence to delineate the peripheral trigeminal nerve. To the best of our knowledge, this is the first application of the Dixon method in post-traumatic trigeminal neuropathy MR neurography studies. Dixon techniques are among a suite of methods used to suppress the signal of fat in MRI. The fat suppression T2-weighted image achieves high-contrast by suppressing the signal intensity of surrounding tissues, including fat, and increasing the T2 contrast of the endoneural fluid inside the peripheral nerve. Moreover, it provides high-resolution and wide applicability to a range of musculoskeletal MR images, making it one of the most preferred sequences in MR neurography [26]. The Dixon techniques display notable advantages compared to other fat suppression techniques. First, they provide homogenous and reliable fat suppression. A significant number of studies suggest that the Dixon technique even achieves fat suppression in areas where other fat-suppression techniques fail for technical reasons [27–29]. Unlike the selective chemical shift fat suppression method, it is insensitive to local magnetic field inhomogeneity [26]. Secondly, it harmonizes with all types of sequences and different types of weightings [30]. A Flex sequence uses a dual echo fat-water separation technology to provide robust and homogeneous fat-suppressed images and can be used with a fast triple echo selection for significant scan time reduction. Enhanced uniformity and control of fat water swaps allow a large field of view and off-center imaging where uniformity is a challenge. However, metal artifacts still pose a challenge to Dixon techniques, especially in the case of large materials such as metallic prostheses [26]. Dental implants, which were a major cause of trigeminal nerve trauma in this study, could thus turn out to be a potential obstacle. To compensate, a 3D STIR sequence was applied as a supportive sequence because it is known for being uniquely independent of precession frequency and field inhomogeneities, even in the presence of metallic subjects [31]. Combining sequences such as slice-encoding metal artifact correction (SEMAC) sequence which is deliberately designed for reducing adverse effects of metal artifacts might be helpful in following the course of the targeted nerve [32]. However, as for this study, T2 FLEX or 3D STIR sequences are deemed more suitable for discriminating the trauma’s distinguishing factor, aSNR, and aNMCNR. Hence, they are relatively more aligned with our purpose. These mutually complementary sequences successfully depicted the nerve morphology, visualized the courses of nerves, and discriminated signal intensity values for traumatic mandibular nerves (Fig. 3A-E). Manual nerve delineation of targeted nerves was agreed among readers (H.W.Y & T.K.O) with ICC of 0.81 for normal side and 0.82 for trauma side; such a result implies more than substantial agreement according to Landis and Koch [33]. In some severe cases, unique radiologic signs of trauma such as ‘triple B sign’ (progressive nerve signal change from ‘bright to black to bright’ across the trauma site) was occasionally depicted via MIP (Fig. 3F) [9, 34]. Xia et al [35]. also excellently implemented post-surgical nerve pathways using Maximum Intensity Projection (MIP) in their study, matching patient symptoms with nerve morphology. However, due to the relatively small sample size, further studies may be needed to systematize and quantify nerve regeneration or the extent of residual trauma.

As mentioned above, while the Dixon and STIR methods have significantly contributed to nerve imaging in T2 MR imaging, efforts are now focused on applying novel techniques like T2 mapping. T2 mapping, offering substantial utility for quantitative measurement in analyzing nerve pathology such as of the extensively researched median nerve [36], is expected to extend its application to future studies, potentially including the mandibular nerve. A diffusion-weighted imaging (DWI) sequence was also considered for application due to of its unique ability to analyze the macro and microscopic structure of peripheral nerves. Moreover, the PROPELLER sequence is considered especially robust in eliminating region-specific artifacts [15]. These two factors indeed helped in selecting the axial ROI corresponding to the T2 coronal section image in this study. In the DWI sequence, while ADC could delineate lesion boundaries by highlighting areas with altered diffusion characteristics, it was unable to discriminate traumatic nerve injury. It has been speculated that such results could be simply due to the limited sample size, or they may be attributed to the low spatial resolution and geometric distortion that frequently occur at the extremities of the human body [9]. Meanwhile, DWI sequencing involves a heterogeneity of algorithms and scanning parameters which renders the reliability and repeatability for routine clinical usage uncertain [19, 37].

Unlike most previous studies of trigeminal nerve injuries, the present study did not confirm the statistical significance of the cross-sectional area of nerve between pathologic nerve and contra-lateral normal side [22]. Several studies of non-trigeminal nerve neuropathy also seem to concur with the idea of increase in nerve caliber and its association with extrinsic injuries [38, 39]. The above studies consider the response of peripheral nerve to compression injury as ongoing degenerative demyelination and compensatory remyelination, which in turn lead to increased myelin sheath thickness and overall increase of nerve diameter, ultimately forming so-called pseudoneuroma. However, the exact pathophysiologic mechanisms underlying nerve caliber increase remains debatable and ambiguous [40, 41]. Moreover, a significant number of studies suggest nerve caliber is unreliable as a MR neurography biomarker. In their prospective study, Kronlage et al. [42] recruited sixty individuals in order to evaluate demographic determinants of nerve caliber and T2 relaxometry. While T2 relaxometry values were independent from demographic components, nerve caliber increased with weight, BMI (body mass index), and height of subjects, such positive correlations being supported by multiple ultrasound studies [43–46]. In accord with the above-mentioned authors, we recommend demographic variables be meticulously controlled with respect to nerve caliber in future studies.

Our study has several limitations. First, being preliminary research, only double significant values (FSNR, FNMCNR) could be derived. This may be a consequence of the small number of study subjects, which is in turn another limiting factor. Secondly, the MRI timing after traumatic events varied due to the nature of the disease. Patients who underwent traumatic injury of trigeminal peripheral nerve usually wait a significant amount of time before visiting a professional department for neurologic evaluation and are often treated conservatively with routine anti-inflammatory medications without proper diagnosis, expecting spontaneous recovery. In our study, average elapsed time from trauma to MR taking was 13.393 months. This may affect diagnostic accuracy, as some researchers have found inconsistencies in MR neurography-derived T2 signal intensity. Chhabra et al. observed some prolonged T2 signal abnormalities in spite of clinical improvement of nerve injury [8]. Husarik et al. [47] pointed out the pitfalls of T2 signal intensity detection among asymptomatic healthy volunteers. Lastly, In MRI imaging, the lower jaw poses a disadvantage due to motion artifacts caused by breathing or swallowing. As a result of such limitation, the data quality of our study may have been inadvertently affected. In this study, however, T2-weighted images were taken using the faster scan technique (FLEX sequence) and DWI was taken using the PROPELLER technique to minimize the impact of motion.

## Conclusions

To the best of our knowledge, this study is the first to identify FSNR and FNMCNR as discriminators for traumatized mandibular nerve based on a novel combination of three different MRI sequences and mixed model analysis. Based on our results, we expect to create a more sophisticated diagnostic model built on multiple variables and large, well-controlled cohorts in further research. Furthermore, MR neurography and deep learning-based research may lead to an auto-detecting diagnostic model for traumatic nerve injury. Such a diagnostic model would enable the establishment of novel clinical guidelines for diagnosing peripheral trigeminal nerve injury and a tangible means of addressing related medico-legal controversies.

## Electronic supplementary material

Below is the link to the electronic supplementary material.

Supplementary Material 1

## Data Availability

In some cases, authors did not generate or use any data to prepare their paper, or are unable to share their data (e.g. the data is confidential or they do not have necessary permissions). In other cases, research data is available upon request. To request the data, contact the corresponding author of the article.

## Abbreviations

ADC: apparent diffusion coefficient
AREA: area of cross-sectional nerve
AUC: area under the receiver operating characteristic curve
DWI: diffusion weighted imaging
FOV: field of view
FNMCNR: 3D STIR apparent nerve-muscle contrast to noise ratio
FSNR: T2 Flex apparent signal to noise ratio
IAN: inferior alveolar nerve
IASP: International Association for the Study of Pain
ICC: intraclass correlation coefficient
LN: lingual nerve
MRCS: Medical Research Council Scale
NSTs: clinical neurosensory tests
PROPELLER: periodically rotating overlapping parallel lines with enhanced reconstruction
QST: quantitative sensory test
ROI: region of interest
SNMCNR: 3D STIR apparent nerve-muscle contrast to noise ratio
SSNR: 3D STIR apparent signal to noise ratio
STIR: short tau inversion recovery
TE: time to echo
TI: time to inversion
TR: repetition time
