# Correction: Assessment of traumatic mandibular nerve using MR neurography sequence: a preliminary study

**DOI:** 10.1186/s12903-024-04769-7

**Published:** 2024-08-23

**Authors:** Hyunwoo Yang, Nak-hoon Son, Dongwook Kim, Jae-Hee Chun, Jin Sung Kim, Tae Kyung Oh, Minwook Lee, Hyung Jun Kim

**Affiliations:** 1 Department of Oral and Maxillofacial Surgery, Yonsei University College of Dentistry, 50-1 Yonsei-ro, Seodaemun-gu, Seoul, 03722 Republic of Korea; 2 Department of Statistics, Keimyung University, Daegu, Republic of Korea; 3 Department of Radiation Oncology, Yonsei Cancer Center, Yonsei University College of Medicine, Seoul, Republic of Korea; 4 Department of Radiology, Yonsei University College of Medicine, Seoul, Republic of Korea

**Correction to: BMC Oral Health (2024) 24:750**

10.1186/s12903-024-04514-0

In this article [1], the author would like to add additional Funding number “6-2022-0016”. The revised Funding is given below.

**Funding**

This study was supported by the Yonsei University College of Dentistry Fund (2-2020-0019) and (6-2022-0016).
