# Correction to: Assessment of traumatic mandibular nerve using MR neurography sequence: a preliminary study

**DOI:** 10.1186/s12903-025-05832-7

**Published:** 2025-03-29

**Authors:** Hyunwoo Yang, Nak-hoon Son, Dongwook Kim, Jae-Hee Chun, Jin Sung Kim, Tae Kyung Oh, Minwook Lee, Hyung Jun Kim

**Affiliations:** 1 Department of Oral and Maxillofacial Surgery, Yonsei University College of Dentistry, 50-1 Yonsei-ro, Seodaemun-gu, Seoul, 03722 Republic of Korea; 2 Department of Statistics, Keimyung University, Daegu, Republic of Korea; 3 Department of Radiation Oncology, Yonsei Cancer Center, Yonsei University College of Medicine, Seoul, Republic of Korea; 4 Department of Radiology, Yonsei University College of Medicine, Seoul, Republic of Korea

**Correction to: BMC Oral Health (2024) 24:750**

10.1186/s12903-024-04514-0

In this article [1], the author would like to add an additional Funding number “6-2022-0016” and to correct the existing funding number from 2-2020-0019 to 6-2020-0021. Both the incorrect and corrected Funding sections are given below.

Incorrect funding section

This study was supported by the Yonsei University College of Dentistry Fund (2-2020-0019) and (6-2022-0016).

Corrected funding section

This study was supported by the Yonsei University College of Dentistry Fund (6-2020-0021) and (6-2022-0016).

## References

[1] Yang H, Son NH, Kim D, Chun JH, Kim JS, Oh TK, Lee M, Kim HJ. Assessment of traumatic mandibular nerve using MR neurography sequence: a preliminary study. BMC oral health. 2024;24(1):750. 10.1186/s12903-024-04514-0 PMC11214249 38943102

