# Supplementary material for: Assessment of traumatic mandibular nerve using MR neurography sequence: a preliminary study
Source: BMC Oral Health. 2024 Jun 28;24:750. doi: 10.1186/s12903-024-04514-0 (PMC11214249; doi:10.1186/s12903-024-04514-0)
Supplement: Supplementary file 1 — Supplementary Material 1 [file 12903_2024_4514_MOESM1_ESM.docx]

**Supplemental material for Assessment of mandibular nerve with MR neurography sequence**

| Sex | Age | MRCS | Severity | Site | traumatic nerve | Type of neuropathic pain | Event of trauma | The duration from  injury to MRI scan  (months) |
| --- | --- | --- | --- | --- | --- | --- | --- | --- |
| F | 66 | S1 | severe | Rt. | Rt.IAN | hyperalgesia | #47 implantation | 1.2 |
| F | 79 | S1 | severe | Lt. | Lt.IAN | hyperalgesia | #36 Extraction & immediate implantation with bone graft | 6.1 |
| F | 74 | S1 | severe | Lt. | Lt.IAN | hyperalgesia, allodynia | #36, 37 implantation | 7.6 |
| F | 57 | S1 | severe | Lt. | Lt.IAN | hyperalgesia, allodynia | #36, 37 implantation | 1.5 |
| F | 28 | S2+ | moderate | Rt. | Rt.LN | hyperesthesia | #48 implantation | 60.9 |
| F | 61 | S1 | severe | Rt. | Rt.IAN | hyperalgesia, dysesthesia | #45, 46, 47 implantation | 4.6 |
| F | 62 | S2+ | moderate | Rt. | Rt.IAN | hyperalgesia | #47 explantation | 9.4 |
| F | 50 | S1 | severe | Rt. | Rt.IAN | hyperalgesia | #40 decortication & bone graft | 31.5 |
| F | 60 | S2+ | moderate | Rt. | Rt.IAN | hyperalgesia | #47 extraction & immediate implantation | 16 |
| F | 67 | S1 | severe | Rt. | Rt.IAN | hyperalgesia | #45, 47 extraction & immediate implantation | 0.9 |
| F | 21 | S2+ | moderate | Lt. | Lt.LN | hyperalgesia | #38 extraction | 15 |
| F | 22 | S3+ | mild | Lt. | Lt.LN | hyperalgesia | #37 implantation & explantation | 8.6 |
| F | 47 | S3+ | mild | Rt. | Rt.IAN | hyperalgesia | #46, 47 implantation | 1 |
| F | 63 | S1 | severe | Rt. | Rt.IAN | hyperalgesia, paresthesia | #46, 47 implant with bone graft | 24.4 |
| F | 49 | S2 | moderate | Rt. | Rt.IAN | hyperesthesia, paresthesia | #44, 45, 46 implantation | 16.6 |
| F | 70 | S2+ | moderate | Rt. | Rt.IAN | hyperesthesia | #46 implantation | 3.7 |
| F | 69 | S3 | mild | Lt. | Lt.IAN | none other than hypoesthesia | #37 implantation | 1.3 |
| M | 62 | S1 | severe | Lt. | Lt.IAN | hyperalgesia, allodynia | #35, 36, 37 implantation | 73 |
| M | 33 | S1 | severe | Rt. | Rt.LN | hyperalgesia | #48 Ext. | 5.1 |
| F | 52 | S1 | severe | Lt. | Lt.IAN | hyperalgesia, paresthesia | #35, 36, 37 implantation & explantation | 0.3 |
| F | 56 | S3+ | mild | Rt. | Rt.IAN | none other than hypoesthesia | #46 implantation | 1.2 |
| M | 59 | S3 | mild | Lt. | Lt.IAN | none other than hypoesthesia | #37 Extraction & immediate implantation | 5.3 |
| F | 59 | S1 | severe | Rt. | Rt.IAN & LN | hyperalgesia | #48 Extraction | 38.4 |
| M | 65 | S1 | severe | Rt. | Rt.IAN | hyperalgesia, allodynia | #46 implantation | 13.3 |
| F | 40 | S2 | moderate | Rt. | Rt.LN | hyperalgesia, paresthesia | #48 Extraction | 2.8 |
| F | 77 | S1 | severe | Lt. | IAN | hyperalgesia | #36 implantation & explantation | 4.7 |

**Supplementary Table 1**. Clinico-pathologic information of enrolled patients; IAN: inferior alveolar nerve; LN: lingual nerve; Rt.: right; Lt.: left.

| Severity vs | normal | p-value | trauma | p-value | Fisher’s Z Test |
| --- | --- | --- | --- | --- | --- |
| FSNR | -0.32089 | 0.1100 | -0.12975 | 0.5276 | 0.4930 |
| SSNR | -0.00059 | 0.9977 | 0.22904 | 0.2604 | 0.4279 |
| ADC | 0.13028 | 0.5259 | 0.05938 | 0.7733 | 0.8082 |
| Area | 0.14183 | 0.4895 | 0.13688 | 0.5049 | 0.9863 |
| FNMCNR | -0.28876 | 0.2774 | -0.21642 | 0.4892 | 0.4798 |
| SNMCNR | -0.25465 | 0.2428 | -0.20411 | 0.3998 | 0.4441 |

**Supplementary Table 2**. Correlation analysis between symptom severity and imaging metrics via Pearson’s Correlation coefficient; FSNR: T2 Flex apparent signal to noise ratio; SSNR : 3D STIR apparent signal to noise ratio ; ADC : Apparent diffusion coefficient; Area : Area of cross sectional nerve; FNMCNR : T2 Flex apparent nerve-muscle contrast to noise ratio; SNMCNR : 3D STIR apparent nerve-muscle contrast to noise ratio

| Severity vs | normal | p-value | trauma | p-value | Fisher’s Z Test |
| --- | --- | --- | --- | --- | --- |
| FSNR | -0.35505 | 0.0751 | -0.00447 | 0.9827 | 0.2136 |
| SSNR | 0.02561 | 0.9012 | 0.32772 | 0.1022 | 0.2859 |
| ADC | 0.07838 | 0.7035 | 0.01787 | 0.9309 | 0.8370 |
| Area | 0.07735 | 0.7072 | 0.02269 | 0.9124 | 0.8525 |
| FNMCNR | -0.32472 | 0.0978 | -0.01068 | 0.8996 | 0.2245 |
| SNMCNR | -0.31404 | 0.1142 | -0.10151 | 0.8511 | 0.1983 |

**Supplementary Table 3**. Correlation analysis between VAS Scale and imaging metrics via Spearman’s Correlation coefficient; FSNR: T2 Flex apparent signal to noise ratio; SSNR : 3D STIR apparent signal to noise ratio ; ADC : Apparent diffusion coefficient; Area : Area of cross sectional nerve; FNMCNR : T2 Flex apparent nerve-muscle contrast to noise ratio; SNMCNR : 3D STIR apparent nerve-muscle contrast to noise ratio

| Severity vs | Normal | Trauma |
| --- | --- | --- |
| FSI | 0.2437 | 0.5785 |
| SSI | 0.9689 | 0.4428 |
| ADC | 0.2892 | 0.4746 |
| Area | 0.2940 | 0.2971 |
| FNMCNR | 0.2564 | 0.6216 |
| SNMCNR | 0.2613 | 0.6168 |

**Supplementary Table 4**. Analysis of variance (ANOVA) analyzing no average difference between symptom severity and imaging metrics; FSNR: T2 Flex apparent signal to noise ratio; SSNR: 3D STIR apparent signal to noise ratio; ADC: Apparent diffusion coefficient; Area: Area of cross sectional nerve; FNMCNR: T2 Flex apparent nerve-muscle contrast to noise ratio; SNMCNR: 3D STIR apparent nerve-muscle contrast to noise ratio

| Severity vs | Normal | Trauma |
| --- | --- | --- |
| FSI | 0.3724 | 0.9987 |
| SSI | 0.6154 | 0.4691 |
| ADC | 0.2307 | 0.4845 |
| Area | 0.2315 | 0.3582 |
| FNMCNR | 0.4241 | 0.9789 |
| SNMCNR | 0.4109 | 0.9699 |

**Supplementary Table 5**. Kruskal-Walis test analyzing no average difference between symptom severity and imaging metrics; FSNR: T2 Flex apparent signal to noise ratio; SSNR: 3D STIR apparent signal to noise ratio; ADC: Apparent diffusion coefficient; Area: Area of cross sectional nerve; FNMCNR: T2 Flex apparent nerve-muscle contrast to noise ratio; SNMCNR: 3D STIR apparent nerve-muscle contrast to noise ratio
